# Emerging RAS superfamily conditions involving GTPase function

**DOI:** 10.1371/journal.pgen.1007870

**Published:** 2019-02-14

**Authors:** Joseph T. C. Shieh

**Affiliations:** Division of Medical Genetics, Department of Pediatrics, Institute for Human Genetics, University of California San Francisco, UCSF Benioff Children's Hospital, San Francisco, California, United States of America; The University of North Carolina at Chapel Hill, UNITED STATES

Mutations in Ras genes lead to several germline genetic conditions with diverse neurodevelopmental and organ system effects. Guanosine triphosphate (GTP)ase activity [1] is a common theme for the classic RAS proteins (e.g., KRAS, HRAS) and of recent RASopathy-associated proteins (e.g., RIT1, [2]). Hiatt and colleagues describe for the first time clinically important variants in *RALA*, a small GTPase (Ras-related protein Ral-A [3]), in individuals with neurodevelopmental conditions [4]. Using exome and genome sequencing, trio analysis for de novo variants, and data sharing through Genematcher [5], the authors describe eleven examples of *RALA* variants associated with a range of clinical findings. The gene variants affected amino acid residues that form the GTP/guanosine diphosphate (GDP) binding region of RALA and decreased GTP hydrolysis in molecular studies. Another small RAS-like GTPase, ARF1, was also recently implicated in a newly recognized neurodevelopmental condition [6], and the pattern of *ARF1* alteration has molecular similarities to *RALA*. For example, the p.K128 residue described by Hiatt and Neu (altered by variants in two individuals) affecting the GTPase active site of RALA was altered by de novo missense alteration in the *ARF1*-neurodevelopmental condition (affecting the equivalent p.K127 residue of ARF1). Examination of control population database variation [7,8] demonstrates variation is depleted in the GTP/GDP-binding region of ARF1 and RALA [4,6]. This suggests a potential logical pattern in human variation and phenotype, in which alteration in GTP/GDP function may lead to disease. If other critical proteins like these are locally intolerant to variation, a resulting missense depleted region (MDR) may be seen in population genomic data [6,9,10]. Ge and colleagues as well as others have demonstrated that localized missense depletion is a useful parameter to consider in analyzing exome data. With new gnomAD data and other population databases, it will also be increasingly important to assess variant occurrence patterns and tolerance to variation. With future studies, we may find out more about how individual GTPase protein function leads to phenotypic features. Hiatt and colleagues, in particular, discuss how RALA effector binding, assessed using an enzyme-linked immunosorbent assay (ELISA), was variable among the RALA variants tested, whereas GTPase activity was consistently diminished. The downstream effects of individual variants could be complex. Both detailed molecular studies and evaluation of clinical and population data will be important in future studies, particularly because small RAS-like GTPases seem to be implicated recurrently in Mendelian conditions. If the key aspects of these RAS superfamily pathways can be understood and targeted [11–14], this may lead to better strategies to modulate these critical pathways.

**Fig 1:**
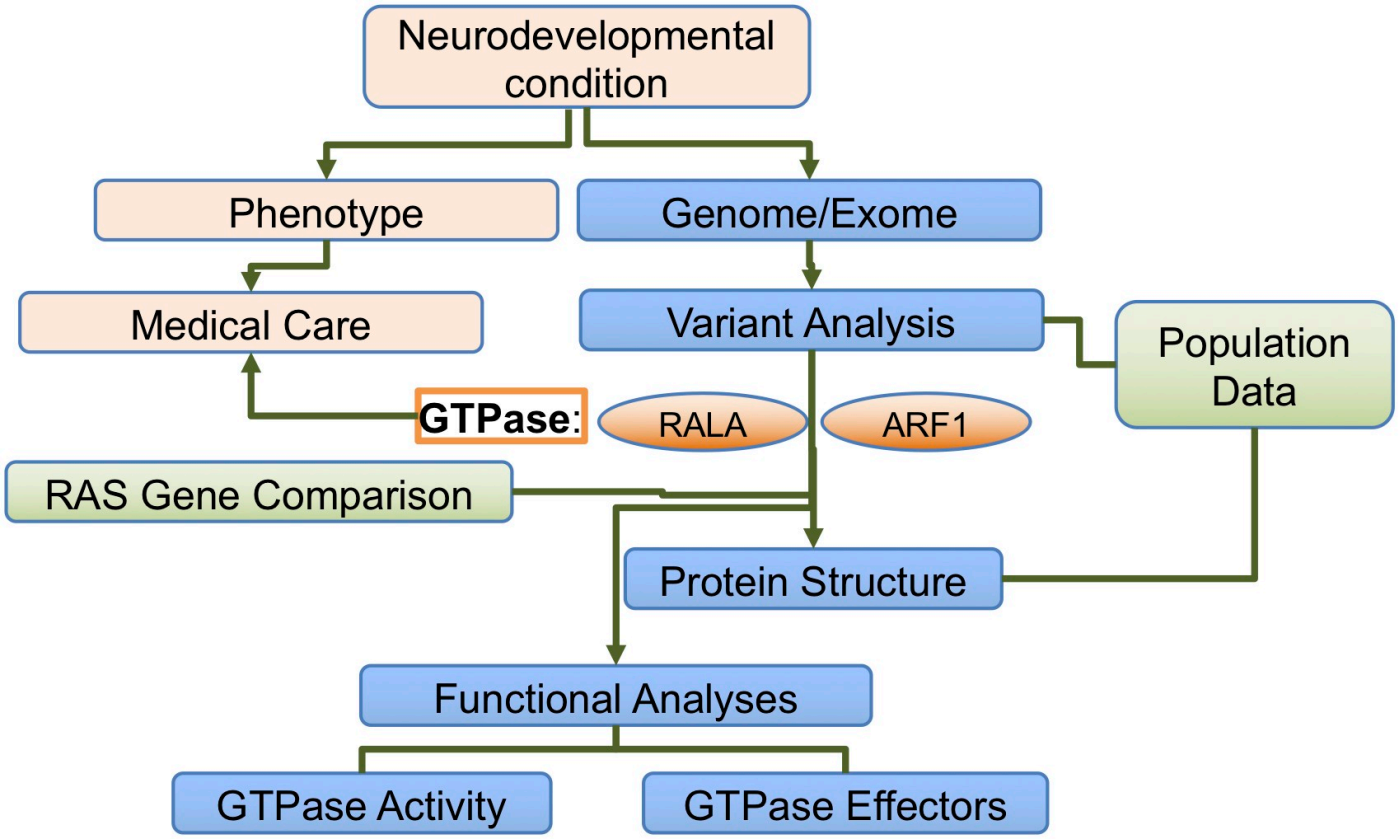
Flowchart. From bedside to bench and back with RAS-superfamily genes RALA and ARF1.

**Fig 2:**
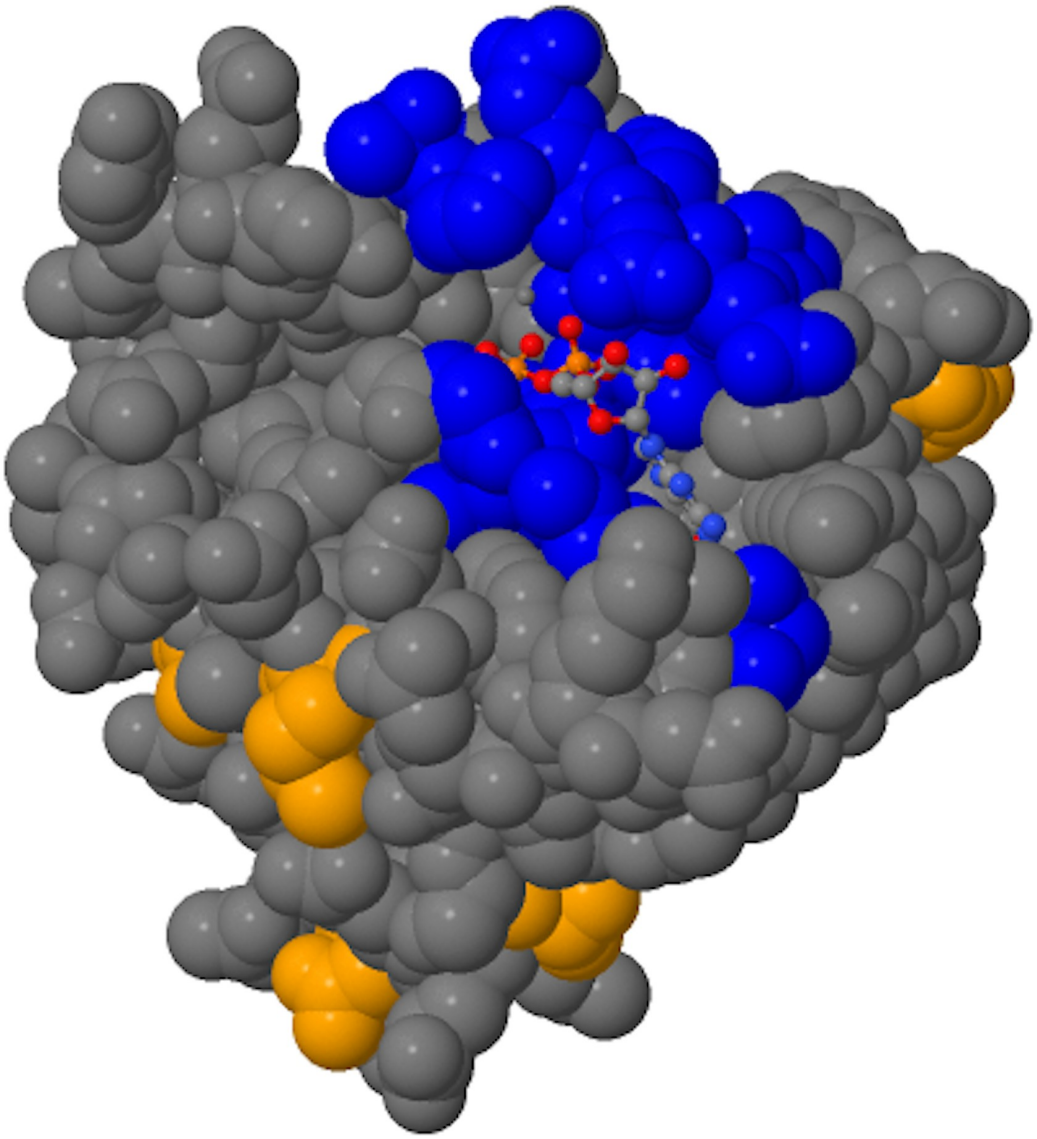
The Ras-superfamily member RALA. Residues of the GTPase site are shown in blue color and population variants in tan color using JSmol (credit: Jiyoo Chang).

The recent findings with RAS-related genes in human genetics (Fig 1) emphasize several salient points. First, conserved RAS-related genes may have important genotype–phenotype correlations. Second, sequencing results are especially informative when coupled with the network of patients and healthcare providers and rich individual-level data. And third, RAS-related gene variants can impact relevant molecular pathways in development. The discovery of these new gene–disease associations bring up a question: What is a Rasopathy? Many of the previously described human conditions involving RAS genes involve the RAS-MAPK pathway, and activation of the pathway is thought to lead to the classic Rasopathies. Some of these pathways have been characterized in detail, and this has led to the development of molecular agents targeting the RAS-MAPK pathway. As suggested by the papers by Hiatt and colleagues (Fig 2) and Ge and colleagues, there is a broader group of highly conserved RAS-superfamily genes that could involve additional molecular mechanisms and pathways. Future studies on potential of loss of function mutations in RAS genes may be informative, because these may represent a different mechanism of disease than classic RAS-MAPK pathway alterations. Hiatt and colleagues show that GTPase activity is decreased with the missense variants, and the truncation mutation, presumably involving resulting in loss of GTPase activity, would also support loss of function. ARF1 also may be decreased in expression in the ARF1-neurologic phenotype (Ge and colleagues). Interestingly, however, is the possibility that there may be gain of function in other molecular aspects of RALA function, such as binding to RALA effectors. Further studies that examine the net effect on the relevant molecular pathways will likely be important for the future, particularly if therapeutic targeting of RAS pathways is considered.

When David Smith [15] and colleagues aggregated phenotypic information for his book on human morphogenesis, and when Victor McKusick and colleagues developed the Mendelian inheritance in man reference [16], they established a new age for clinical genetics, in which phenotypic features were recognized and systematically assessed for clinical care. Patterns of findings were helpful for diagnosis and for targeting medical management. When new features of a given clinical entity were noted, the range of phenotypic features were reevaluated, and the condition criteria were refined or expanded. Cardinal phenotypic features and medical constellations continue to be helpful for how we think about Mendelian conditions today. With genetic testing becoming much more common, a combination of phenotyping and genotyping often provides answers in previously undiagnosed conditions. Some clinicians and scientists are lumpers and some are splitters when considering phenotypic categorization of features, however, with genetic data being generated at a more rapid pace, we are faced with the complex task of integrating the genotypic and phenotypic features on a much broader scale. What is the best way of grouping health conditions together? Is it the recognizable physical features that assist with diagnostic testing and management, or is a common genetic pathway that may lead to therapeutic investigation? Both approaches may play an important role. The RASopathies have contributed to a better understanding of disease, because families and clinicians recognize the common occurrence of variation in these genes. The confluence of RAS variant-associated cancers and germline conditions also hints at the complexity of human phenotypic variation. Certain germline RAS medical conditions are associated with an increased chance of cancer, whereas some are not. Currently, it is not clear how to stratify individuals who have a germline genetic condition regarding an increased—but unclear to what degree—risk of cancer. If further exome and genome sequence information are to be used to answer these management questions and other health risks, such information will need to be understood in much more detail. With germline *RALA* and *ARF1* gene variant phenotypes, we should try to understand the scope of developmental effects that are due to these genes. Variants in these genes have primarily neurodevelopmental effects based on our understanding to date, despite the fact that expression is predicted to be widespread in the body. Further assessment of RALA and ARF1 function in brain will be needed to move us forward.
